# Role of primary care for individuals with childhood-onset neurologic conditions

**DOI:** 10.1016/j.hctj.2023.100037

**Published:** 2023-12-29

**Authors:** Miho Osako, Yui Yamaoka, Yoko Mochizuki, Takeo Fujiwara

**Affiliations:** a Department of Neurology, Tokyo Metropolitan Kita Medical and Rehabilitation Center for the Disabled, 1–2-3 Jujodai, Kita-ku, Tokyo 114–0033, Japan; b Department of Global Health Promotion, Tokyo Medical and Dental University, 1–5-45, Yushima, Bunkyo-ku, Tokyo 113–8519, Japan

**Keywords:** Childhood-onset neurological conditions, Pediatric-to-adult health care transition, Primary care, Community- and hospital-based care, Intellectual disabilities

## Abstract

**Background:**

Individuals with childhood-onset neurologic conditions often face challenges in the pediatric-to-adult health care transition (HCT). Furthermore, the importance of implementing primary care is unrecognized. We investigated the situation of adults with childhood-onset neurologic conditions from the perspective of health care professionals (HCPs) in community- and hospital-based primary care practice.

**Design and methods:**

Overall, 1334 HCPs in medical facilities across Tokyo (mainly in Kita, Nerima, and Itabashi Cities) were surveyed regarding their experience caring for adults with childhood-onset neurologic conditions. Snowball sampling was also deployed to enhance the input from various health professions. The questionnaire included quick response codes linked to web-based questionnaires identical to paper-based ones, enabling additional HCPs to answer the questionnaire. The survey included questions about the care provided by HCPs, the perceived challenges and worthwhileness of the care, and their views on HCT.

**Results:**

We collected 276 responses (response rate, 20.7%): 224 by mail and 52 online. In total, 94 HCPs of the respondents (75 doctors, 11 nurses, 5 therapists, 2 care workers, and 1 medical social worker) involved in caring for this population were analyzed. Doctors and nurses managed medical devices, educated patients, and provided consultation and care. Doctors cited the management of comorbidities outside of their expertise and difficulties securing hospitalization during emergencies as barriers to care. HCPs found the valuable opportunities to enrich their clinical experience and long-term relationships with patients worthwhile. HCPs expressed the need for systems that guarantee patient hospitalization and multidisciplinary conferences between HCPs and specialists.

**Conclusion:**

We described the roles of HCPs in community- and hospital-based primary care, which are vital components of HCT for adults with childhood-onset neurologic conditions. Their practice includes multidisciplinary involvement, patient education, and care coordination. For better HCT in this population, efforts are required to enhance HCPs’ capability to respond to patients with disabilities, patients’ multiple comorbidities, and families’ needs.

**Practice implications:**

Further efforts to deepen community-based care are desired to improve HCT for people with childhood-onset neurologic conditions.

## Highlights

• We described the role of primary care in childhood-onset neurologic conditions.
• The care is delivered through community- and hospital-based practice.
• The practice includes multidisciplinary involvement, education, and coordination.
• Efforts to deepen primary care are desired to improve health care transition.

## 1 Introduction

The life expectancy of individuals with childhood-onset conditions has increased with technological progress. Pediatric-to-adult health care transition (HCT) is a process that supports young people with childhood-onset medical conditions to move from family-oriented pediatric practice to disease-oriented adult practice and adapt to adult life.^1^ Structured HCT comprises transition and care policy/guidance, tracking and monitoring, transition readiness, transition planning, transfer of care, and transfer completion.^2^ Studies have shown that structured HCT is related to better population health, patient well-being, cost reduction, and service utilization.^3^, ^4^, ^5^ Professional entities such as the American Academy of Pediatrics, the American Academy of Family Physicians, and the American College of Physicians have advocated for the dedication of more attention and resources to healthcare transition.^6^ However, because individuals with childhood-onset neurological conditions often have intellectual, developmental, and physical disabilities, the transition to an adult department is sometimes complicated.^7^

Since its establishment in 1985, the Tokyo Metropolitan Kita Medical and Rehabilitation Center for the Disabled has been a comprehensive medical facility for people with disabilities. The hospital provides medical care, respite care, and rehabilitation for children and adults with disabilities. The primary disease in many patients is childhood-onset neurological conditions such as cerebral palsy, Down syndrome, or epilepsy syndrome.^8^ The HCT of these individuals includes treatment adjustment, patient education, and promotion of community-based care.^9^ Many patients use visiting doctors and nurse care services and depend on community-based primary care.^8^

The involvement of primary health care professionals (HCPs) in HCT is important for the continuity and accessibility of care,^10^ and the participation of primary care doctors is associated with better outcomes for HCT.^11^, ^12^ Furthermore, primary care is crucial in person-centered care for individuals with disabilities.^13^ However, how HCPs involved in primary care for adults with childhood-onset neurologic conditions recognize their role remains unclear in Japan. Until recently, there was no system of general or family physicians in Japan who could play as gatekeepers of medical practice and Japanese citizens have almost unrestricted access to medical facilities.^14^ Primary care is generally provided in community-based offices such as clinics or small and medium-sized hospitals in Japan. ^15^

We aimed to explore the role of community- and hospital-based primary care for individuals with childhood-onset neurologic conditions. We investigated the perspectives of HCPs who currently provide primary care for adults with childhood-onset neurologic conditions and compared them to make recommendations for better HCT.

## 2 Methods

### 2.1 Development of questionnaire

We developed self-administered structured questionnaires for this study to collect information from various health care workers because there had not been a survey questionnaire that included various professionals (not only doctors and nurses, but also other HCPs) to answer regarding HCT. A pediatrician (YY) and a neurologist (MO) with abundant practice experience in HCT developed a questionnaire and consulted with stakeholders (2 clinic doctors, 4 nurses from visiting nurse stations, and 4 nurses from hospitals) as well.

### 2.2 Study participants

We defined HCPs in this study as persons in all professions involved in health care. We targeted the HCPs involved in community- and hospital-based primary care in this study as follows: i) doctors whose facilities were registered with district medical associations, a branch of Japan Medical Associations, which is a nationwide association of medical doctors in Japan that play a crucial role in community-based primary care^16^ and nurses working for visiting nurse offices. We chose facilities in the Kita City, where our hospital is located, and the Itabashi and Nerima Cities, adjacent to the Kita City. These three municipalities had the largest number of patients visiting our hospital in 2019; ii) doctors with whom we have had medical cooperation, such as doctors who have referred patients to our hospital for consultation or utilization of welfare services within Tokyo; and iii) HCPs working in a medical daycare center for adults with profound and multiple intellectual disabilities within Tokyo certified by the Tokyo Metropolitan Government. We did not limit our target doctors to only pediatrics and internal medicine because we wanted to identify what departments were involved in caring for these patients. Furthermore, snowball sampling was also deployed to investigate perspectives from various health professions. The questionnaire included quick response codes linked to web-based questionnaires identical to paper-based ones, enabling additional HCPs to answer the questionnaire. The participants answered either the paper- or web-based questionnaire.

We distributed questionnaires to health professionals in 1334 medical and welfare facilities in the community; 1086 medical institutions registered with the Japan Medical Associations in Kita, Nerima, and Itabashi Cities; 174 visiting nurse offices located in Kita, Nerima, and Itabashi Cities; 27 medical institutions with medical cooperation with our hospital within Tokyo; and 47 medical daycare centers within Tokyo.

### 2.3 Demographics of HCPs caring for adults with childhood-onset neurological conditions

The questionnaire asked participants about their occupations (medical doctor, nurse, social worker, and others). The number of nurse practitioners in Japan is very small, and so the answer “nurse” here does not assume a nurse practitioner. Nurses can perform nursing care at their own discretion, but all medical treatment is performed under the direction of a medical doctor in Japan. Background specialty (pediatrics, internal medicine, neurology, neurosurgery, surgery, ophthalmology, otolaryngology, dermatology, psychiatry, gynecology, and orthopedics), and type of facility where they were employed (hospital, clinic, visiting nurse station, welfare facility, and government) were also asked. In this study, we explained the definition of “adults with childhood-onset neurological conditions” to mean adult patients aged 18 years or older with neurologic conditions that developed before the age of 18. We listed examples of childhood-onset neurological conditions such as cerebral palsy, epilepsy syndrome, Down syndrome and other chromosomal abnormalities, intellectual disability, and autism spectrum disorder in the questionnaire. Next, we asked whether they are involved in caring for adults with childhood-onset neurological conditions (“yes” or “no”). Those who answered “yes” were asked to answer further questions, including whether they provided home-visit care, the time spent per patient in outpatient or home-visit care, and the number of adult patients with childhood-onset neurological conditions they were currently in charge of. Participants indicated how often and from which specialist their patients were referred on a 5-point scale (1 = never, 2 = seldom, 3 = sometimes, 4 = frequently, and 5 = always), using the following items: “transferred/referred from pediatric specialists in hospitals,” “transferred/referred from adult specialists in hospitals,” “transferred/referred from another primary care doctor,” “patient or family self-referred,” and “since birth or childhood.” In a Likert scale item, Japanese people tend to avoid extreme responses.^17^ Therefore, we dichotomized “frequently” and “always” as “yes,” and indicated the number and percentage (%) of those who answered “yes” as the result.

### 2.4 Care for adults with childhood-onset neurologic conditions in community-based care

To determine the experience of HCPs in community- and hospital-based practice, we examined to what extent HCPs provide care to adults with childhood-onset neurologic conditions for the following items concerning the description of care that HCPs provide: “regular consultation,” “patient education,” “paperwork,” “vaccination,” “symptomatic treatment,” “coordination of care,” “management using medical devices,” “referrals,” “management of psychological symptoms,” “rehabilitation,” and “physical care” using a 5-point Likert scale (1 = strongly disagree to 5 = strongly agree) for each item. We also assessed perceived challenges in the care of adults with childhood-onset neurological conditions in terms of “management of comorbidities outside of the HCP’s specialty/expertise,” “difficulty in communicating with patients,” “undecided emergency policy,” “needs for special attention to family’s feelings,” “lack of information on adult services,” “overwhelmed with the demands of the family,” “difficulty in referral during emergencies,” “difficulty in identifying and coping with family issues,” and “reluctance of families about consulting other hospitals.” Furthermore, we investigated the benefits of assigning a primary care doctor for patients; “accessibility,” “care for all aspects of life,” “family-centered care,” “patient education opportunities,” and “coordinated care.” We dichotomized “strongly agree” and “agree” as “yes.”

### 2.5 HCPs’ opinion on pediatric-to-adult HCT of individuals with childhood-onset neurologic conditions

To determine the roles of HCPs in pediatric-to-adult HCT of individuals with childhood-onset neurologic conditions, we assessed the experience of discussions of HCPs with patients regarding HCT before transfer (“yes” or “no”) and the topics discussed regarding “whether the patient should undergo HCT or not” (in Japan, pediatricians often continue to see adult patients), “difficulty in finding adult practitioners who accept patients undergoing HCT,” and “benefits/challenges of HCT.”

To explore the facilitating factors of HCT in community-based care, we investigated what HCPs find rewarding in the care of adults with childhood-onset neurological conditions using the following items: “experience opportunities with a variety of disease cases including rare diseases,” “patients with childhood-onset neurological conditions often have disabilities, and contact with people with disabilities broadens medical professionals’ perspectives,” “engagement with patients on a long-term basis,” and “be able to develop expertise in social welfare systems.” We also investigated the preferred types of transition from pediatrics to adult health care in terms of “transition to adult care after a certain period of concurrent pediatric and adult care,” “pediatrician as physician in charge and specialized adult departments as needed,” “continuation of pediatric care in adulthood without HCT,” and “transfer to adult care as soon as the patient reaches the age of adulthood (18 years old).” We investigated desired systems that serve HCPs involved in the care of adults with childhood-onset neurological conditions regarding “facilities for emergency hospitalization,” “sharing patients’ medical information,” “specialists to respond to inquiries from primary care doctors,” “multidisciplinary conference,” and “management fee.” We assessed the responses for each item on a 5-point Likert scale (1 = strongly disagree to 5 = strongly agree). We dichotomized “strongly agree” and “agree” as “yes.”

### 2.6 Statistical analysis

We used descriptive statistics throughout the study. First, we described the respondents’ demographics. Second, we described the participants’ experiences caring for adults with childhood-onset neurological conditions. Finally, we described participants’ opinions and desires regarding caring for adults with childhood-onset neurological conditions in community-based care. We conducted the analyses using Stata/IC version 17 (STATA Corp., College Station, TX).

### 2.7 Standard protocol approvals, registrations, and patient consents

This study was approved by two Institutional Review Boards (M2020–168) and (2020–6). The questionnaire had a checkbox for respondents to provide consent to participate in the study. For the electronic surveys, respondents provided consent by marking a checkbox to proceed with further questions. We obtained informed consent from all participants and anonymized their information for analysis.

### 2.8 Data availability

The questionnaires and study data are available upon request from the corresponding author (M.O.).

## 3 Results

### 3.1 Characteristics of participants

We collected 276 responses (response rate, 20.7%): 224 by mail and 52 online including responses that may have been obtained through snowball sampling. Table 1 shows the demographics of the respondents. In total, 94 out of 276 (34.1%) HCPs were involved in caring for adults with childhood-onset neurologic conditions. Most professionals involved in the care were doctors (n = 75, 79.8%), and the respondents involved in the care mainly worked in clinics (n = 63, 67.0%; 62 doctors and 1 nurse).

**Table 1:** Demographics of respondents.

Variables			n (%)	
Involved in the care of adults with childhood-onset neurologic conditions			Yes	No
Total			94 (34.1)	182 (65.9)
Profession	Doctor		75 (34.2)	144 (65.8)
		Hospital	12 (66.7)	6 (33.3)
		Clinic	62 (31)	138 (69)
		Medical daycare	1	0
	Nurse		11 (34.4)	21 (65.6)
		Hospital	1 (33.3)	2 (66.7)
		Clinic	1 (5.6)	17 (94.4)
		Visiting nurse station	7 (87.5)	1 (12.5)
		Medical daycare	2	0
		Government	0	1
	Therapist (physiotherapist or occupational therapist)			
		Visiting nurse station	5	0
	Care worker			
		Medical daycare	2 (50)	2 (50)
	Medical social worker			
		Visiting nurse station	1	0
	Consultation support specialist			
		Medical daycare	0	1
	Care manager			
		Home care support office	0	1
	Life support worker			
		Medical daycare	0	5
	Childminder			
		Medical daycare	0	1
	Administrator		0	7
		Hospital	0	1
		Clinic	0	1
		Visiting nurse station	0	1
		Medical daycare	0	4

Consultation support specialists provide consultation, help people with disabilities receive appropriate welfare services, and connect them with nursing care facilities. Care managers specialize in long-term care insurance and create care plans coordinating with service providers. Life support workers provide nursing care such as meals, bathing, and toileting and support for household chores such as cooking, cleaning, laundry, and money control at facilities for persons with disabilities.

Table 2 presents the care practice delivered by doctors. The most common specialty background among doctors was internal medicine (n = 25), followed by pediatrics (n = 15), otolaryngology (n = 10), neurology (n = 5), and surgery (n = 5). Clinic doctors practiced more home visits than hospital doctors. Regarding referral sources of patients that respondents were assigned, hospital doctors saw most patients since their childhood without referrals (58.3%). In contrast, clinic doctors saw patients with self-referral, implying that patients came to the medical facility independently without referrals (58.1%).

**Table 2:** Background specialty of doctors and care practice provided by doctors classified by institutions for adults with childhood-onset neurologic conditions.

Variables			n (%)		
			Clinics (n = 62)	Hospitals (n = 12)	Medical daycare (n = 1)
Subspecialty					
		Pediatrics	8	6	1
		Internal medicine	23	2	0
		Neurology	4	1	0
		Surgery	4	1	0
		Ophthalmology	4	0	0
		Otolaryngology	10	0	0
		Dermatology	2	0	0
		Psychiatry	5	0	0
		Gynecology	1	0	0
		Orthopedic	1	2	0
Ability to provide home visitation services			27 (43.5)	5 (41.7)	0
Time spent per patients (min) (Clinics n = 59, Hospitals n = 12, Welfare facility n = 1) mean, SD			13.6 ± 8.7	13.8 ± 4.3	20
Number of patients in charge (Clinics n = 55, Hospitals n = 11, Welfare facility n = 1)					
		< 5	44 (80)	5 (45.5)	0
		5–9	1 (1.8)	2 (18.2)	0
		10–19	4 (7.3)	0	0
		≥ 20	6 (10.9)	4 (36.4)	1
Classification of referral sources*					
	Transferred/referred from	Pediatric specialists in hospitals	15 (24.2)	3 (25)	1
		Adult specialists in hospitals	14 (22.6)	1 (8.3)	1
		Other primary care doctor	12 (19.4)	1 (8.3)	0
	Patient or family self-referred		36 (58.1)	2(16.7)	0
	Since birth or childhood		21 (33.9)	7 (58.3)	1

* Participants indicated how often and from which specialist their patients were referred on a 5-point scale (1 = never, 2 = seldom, 3 = sometimes, 4 = frequently, and 5 = always). “Frequently” and “always” are dichotomized as “yes.”

### 3.2 Community- and hospital-based primary care practice for adults with childhood-onset neurologic conditions

**Fig. 1:**
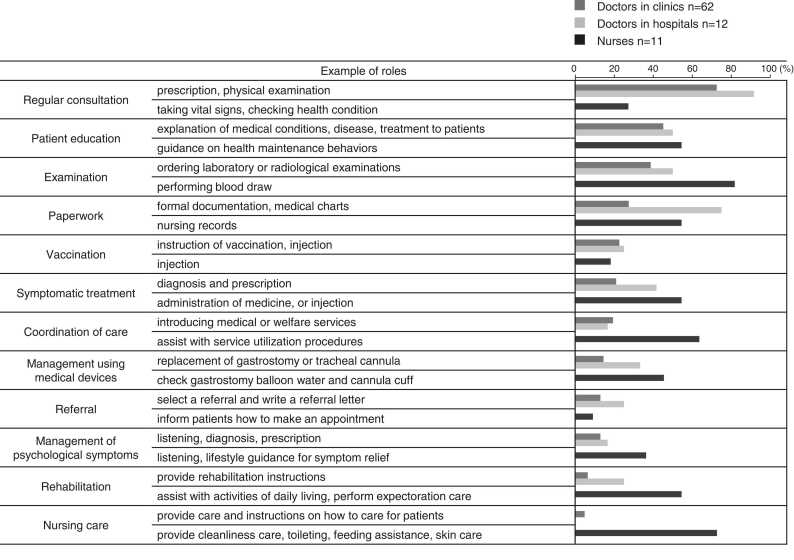
Description of care provided by doctors in clinics or hospitals, and nurses for childhood-onset neurologic conditions. Description of care provided by each profession and example of their roles for childhood-onset neurologic conditions. The health care professionals were asked about their practices for adults with childhood-onset neurologic conditions. Their responses were examined on a 5-point Likert scale (1 = strongly disagree to 5 = strongly agree) and dichotomized as “strongly agree” and “agree” as “yes.”.

Fig. 1 illustrates the care provided by doctors from clinics and hospitals, and nurses and their roles in the care. Hospital and clinic doctors perform paperwork, patient education, and regular consultations. Nurses are highly involved in education, service coordination, device management, and emergency response other than regular duties, which are care and rehabilitation. Table 3 shows the doctors’ practice descriptions summarized by specialty. Pediatricians, internists, and neurologists were involved in many areas of care.

**Table 3:** Medical doctors’ practice descriptions summarized by specialty (n [%]).

	Pediatrics			Internal medicine		Neurology		Surgery	
	Clinic (n = 8)	Hospital (n = 6)	Medical daycare (n = 1)	Clinic (n = 23)	Hospital (n = 2)	Clinic (n = 4)	Hospital (n = 1)	Clinic (n = 4)	Hospital (n = 1)
Regular consultation	5 (71.4)	6 (100)	1	18 (78.3)	2	4	1	2 (50)	1
Patient education	3 (42.9)	3 (50)	1	8 (36.4)	2	2 (50)	0	0	0
Examination	2 (25)	2 (33.3)	1	17 (77.3)	2	2 (50)	1	1 (25)	0
Paperwork	3 (37.5)	5 (83.3)	1	6 (26.1)	2	2 (50)	1	1 (25)	0
Vaccination	3 (50)	1 (16.7)	1	8 (36.4)	2	1 (25)	0	1 (25)	0
Symptomatic treatment	2 (25)	3 (50)	1	7 (33.3)	2	1 (25)	0	1 (25)	0
Coordination of care	2 (25)	1 (16.7)	1	6 (27.3)	1 (50)	2 (50)	0	0	0
Management using medical device	2 (25)	3 (50)	1	6 (27.3)	1 (50)	0	0	1 (25)	0
Referral	2 (25)	1 (16.7)	1	4 (17.4)	2	1 (25)	0	0	0
Management of psychological symptoms	2 (25)	2 (33.3)	1	1 (4.5)	0	0	0	0	0
Rehabilitation	2 (25)	1 (16.7)	1	1 (4.5)	1 (50)	0	0	0	0
Nursing care	3 (37.5)	0	1	1 (4.5)	0	0	0	0	0

Table 3 continued							
	**Ophthalmology**	**Otolaryngology**	**Dermatology**	**Psychiatry**	**Gynecology**	**Orthopedic**	
	**Clinic** **(n = 4)**	**Clinic** **(n = 10)**	**Clinic** **(n = 2)**	**Clinic** **(n = 5)**	**Clinic** **(n = 1)**	**Clinic** **(n = 1)**	**Hospital** **(n = 2)**

Regular consultation	3 (75)	6 (60)	2	5	0	1	1 (50)
Patient education	3 (75)	4 (44.4)	1 (50)	5	0	1	1 (50)
Examination	0	0	1 (50)	0	0	1	1 (50)
Paperwork	0	0	0	5	0	0	1 (50)
Vaccination	0	0	0	0	0	0	0
Symptomatic treatment	0	1 (11.1)	0	1 (20)	0	0	0
Coordination of care	0	0	1 (50)	1 (20)	0	0	0
Management using medical device	0	0	0	0	0	0	0
Referral	0	0	0	1 (20)	0	0	0
Management of psychological symptoms	0	0	0	5	0	0	0
Rehabilitation	0	0	0	0	0	0	1 (50)
Nursing care	0	0	0	0	0	0	0

Participants were asked what extent they provide care to adults with childhood-onset neurologic conditions using a 5-point Likert scale (1 = strongly disagree to 5 = strongly agree). “Strongly agree” and “agree” were dichotomized as “yes.”

The challenges in caring for adults with childhood-onset neurologic conditions perceived by each profession are described in Table 4. The most frequent challenge among doctors was “managing comorbidities outside of their specialty/expertise,” followed by “communicating with patients” among clinic doctors and “difficulty with referral in case of emergency” among hospital doctors. Nurses answered that the two main challenges were “the need for special attention to the family’s feelings” and “overwhelmed with the demands from the family.”

**Table 4:** Challenges in the care of adults with childhood-onset neurologic conditions from health care professionals’ perspectives.

Variables	n (%)						
	Doctors			Nurses (n = 11)	Therapists (n = 5)	Care worker (n = 2)	Medical social worker (n = 1)
	Clinics (n = 62)	Hospitals (n = 12)	Medical daycare (n = 1)				
Management of comorbidities outside of the HCP’s specialty/expertise	42 (67.7)	10 (8.3)	1	2 (18.2)	1 (20)	1 (50)	0
Difficulty in communicating with patients	32 (51.6)	2 (16.7)	0	0	2 (40)	2 (100)	0
Undecided emergency policy	27 (43.5)	3 (25)	0	4 (36.4)	1 (20)	2 (100)	0
Need for special attention to family’s feelings	24 (38.7)	4 (33.3)	0	9 (81.8)	3 (60)	2 (100)	1
Lack of information on adult services	23 (37.1)	6 (0.5)	0	4 (36.4)	2 (40)	2 (100)	1
Overwhelmed with the demands of the family	22 (35.5)	6 (0.5)	0	9 (81.8)	4 (80)	1 (50)	0
Difficulty in referral during emergencies	21 (33.9)	9 (75)	0	7 (63.6)	0	1 (50)	1
Difficulty in identifying and coping with family issues	16 (25.8)	2 (16.7)	0	6 (54.5)	3 (60)	1 (50)	1
Reluctance of families about consulting other professionals	9 (14.5)	3 (25)	0	5 (45.5)	1 (20)	1 (50)	0

HCP: health care professional, Challenges in the care perceived by each profession. The HCPs were asked about the perceived challenges in caring for adults with childhood-onset neurologic conditions. Their responses were examined on a 5-point Likert scale (1 = strongly disagree to 5 = strongly agree) and dichotomized as “strongly agree” and “agree” as “yes.”

Patients’ benefits of having primary care doctors, as perceived by HCPs, are shown in Table 5. Clinic and hospital doctors, nurses, and therapists appreciated the family-centered care provided by primary care doctors and their accessibility.

**Table 5:** Patients’ benefits of assigning primary care doctor from health care professionals' perspectives.

Variables	n (%)						
	Doctors			Nurses (n = 11)	Therapists (n = 5)	Care worker (n = 2)	Medical social worker (n = 1)
	Clinics (n = 62)	Hospitals (n = 12)	Medical daycare (n = 1)				
Accessibility	59 (95.2)	9 (75.0)	1	10 (90.9)	5 (100)	1 (50)	1
Care for all aspects of life	33 (53.2)	6 (50.0)	0	9 (81.8)	4 (80)	1 (50)	0
Family-centered care	31 (50.0)	4 (33.3)	0	9 (81.8)	3 (60)	1 (50)	0
Patient education opportunities	17 (27.4)	1 (8.3)	0	4 (36.4)	3 (60)	0	0
Coordinated care	29 (46.8)	7 (58.3)	0	7 (63.6)	4 (80)	1 (50)	0

Participants were asked about the benefits of assigning a primary care doctor for patients using a 5-point Likert scale (1 = strongly disagree to 5 = strongly agree) for each item. “Strongly agree” and “agree” were dichotomized as “yes.”

### 3.3 Pediatric-to-adult HCT for individuals with childhood-onset neurologic conditions in community- and hospital-based primary care

Table 6 shows HCPs’ experience and opinions about HCT for individuals with childhood-onset neurologic conditions. Regarding the experience of discussing with patients about HCT, hospital doctors (50.0%) were more likely to report having conversations with patients than clinic doctors (33.9%). Nurses (72.7%) and therapists (100%) were more likely to report having these discussions than doctors. Regarding what HCPs find worthwhile in caring for patients with childhood-onset neurologic conditions, clinic (n = 35, 56.5%) and hospital (n = 6, 50%) doctors cited “experience opportunities with a variety of disease cases including rare diseases.” In contrast, nurses thought “engagement with patients on a long-term basis” was most rewarding (n = 9, 81.8%). Therapists found it rewarding in terms of increasing their professional experience and knowledge (n = 5, 100%), as well as their long-term relationship with patients (n = 5, 100%) and the impact on their perspectives on life (n = 5, 100%). Regarding the preferred types of transition, clinic and hospital doctors and nurses preferred “transition to adult care after a certain period of concurrent pediatric and adult care” the most, while therapists preferred “pediatrician as a physician in charge and specialized adult departments as needed” the most.

**Table 6:** Experience of discussion with patients about health care transition and facilitators of transition in community- and hospital-based primary care from health care professionals’ perspectives.

Variables	n (%)						
	Doctors			Nurses (n = 11)	Therapists (n = 5)	Care worker (n = 2)	Medical social worker (n = 1)
	Clinics (n = 62)	Hospitals (n = 12)	Medical daycare (n = 1)				
Experience of discussions with patients about HCT	21 (33.9)	6 (50.0)	1	8 (72.7)	5 (100)	1 (50)	1
Topics discussed with patients and families regarding transition							
Whether the patient should undergo HCT or not	12 (57.1)	5 (83.3)	0	4 (50.0)	2 (40)	0	0
Difficulty in finding adult practitioners who accept patients undergoing HCT	11 (52.4)	6 (100)	1	7 (87.5)	3 (60)	1 (50)	1
Benefits/challenges of HCT	8 (38.1)	4 (66.7)	1	5 (62.5)	4 (80)	1 (50)	1
Facilitators of HCT in community- and hospital-based primary care from HCPs' perspectives							
What HCPs find rewarding in caring for patients with childhood-onset neurologic conditions							
Experience opportunities with a variety of disease cases including rare diseases	35 (56.5)	6 (50)	0	7 (63.6)	5 (100)	2 (100)	1
Patients with childhood-onset neurologic conditions often have disabilities, and contact with people with disabilities broadens medical professionals' perspectives	33 (53.2)	3 (25)	0	5 (45.5)	5 (100)	2 (100)	1
Engagement with patients on a long-term basis	27 (43.5)	5 (41.7)	0	9 (81.8)	5 (100)	1 (50)	1
Be able to develop expertise in social welfare systems	24 (38.7)	5 (41.7)	0	5 (45.5)	4 (80)	2 (100)	1
Preferred types of transition from pediatrics to adult health care							
Transition to adult care after a certain period of concurrent pediatric and adult care	40 (64.5)	9 (75)	0	9 (81.8)	3 (60)	2 (100)	0
Pediatrician as the physician in charge and specialized adult departments as needed	30 (48.4)	4 (33.3)	0	6 (54.5)	4 (80)	2 (100)	1
Continuation of pediatric care without HCT	20 (32.3)	1 (8.3)	0	2 (18.2)	2 (40)	1 (50)	0
Transfer to adult care as soon as the patient reaches 18 years old	12 (19.4)	1 (8.3)	1	1 (9.1)	0	1 (50)	0
Desired systems for the care of adults with childhood-onset neurologic conditions in community- and hospital-based primary care							
Facilities for emergency hospitalization	48 (77.4)	10 (88.3)	0	9 (81.8)	4 (80)	2 (100)	1
Sharing patients’ medical information	36 (58.1)	3 (25.0)	1	5 (45.5)	5 (100)	1 (50)	0
Specialists to respond to inquiries from primary care doctors	32 (51.6)	7 (58.3)	0	6 (54.5)	2 (40)	1 (50)	0
Multidisciplinary conference	16 (25.8)	4 (33.3)	0	8 (72.7)	4 (80)	2 (100)	0
Management fee	16 (25.8)	5 (41.7)	0	6 (54.5)	1 (20)	0	0

Regarding experience of discussion with patients about HCT and facilitators of HCT in the community-based care, participants were asked to answer each item on a 5-point Likert scale (1 = strongly disagree to 5 = strongly agree). “Strongly agree” and “agree” were dichotomized as “yes.” HCP: health care professional, HCT: health care transition.

Regarding desired systems to care for adults with childhood-onset neurological conditions in a community-based care system, doctors in clinics (n = 48, 77.4%) and hospitals (n = 10, 88.3%), nurses (n = 9, 81.8%), therapists (n = 4, 80%), and care workers (n = 2, 100%) declared the need for facilities for emergency hospitalization. For doctors working in clinics, sharing patients’ medical information was the second most popular demand (58.1%), followed by a system in which experts will respond to inquiries from primary care doctors (51.6%), which was the second most popular request among hospital doctors (58.3%). More than 70% of nurses and therapists needed multidisciplinary conferences to share information about patients and care management.

## 4 Discussion

This study revealed the experiences and opinions of HCPs currently providing primary care practice for adults with childhood-onset neurological conditions. Although the response rate of 20.7% in this study may seem low, it is justified because the assumption is that the number of adult patients with childhood-onset neurologic conditions is much smaller than the number of patients with other common conditions. For instance, the prevalence of cerebral palsy at birth is 1.6 per 1000 live births.^18^ It is possible that some HCPs who received the questionnaire did not complete it because they had not treated such patients before.

Doctors treating these adult patients in a hospital were more likely to have a pediatric background than those treating in a clinic, and had been seeing these adult patients since childhood. Therefore, many hospital doctors would continue to see patients from childhood as primary care doctors without transitioning from pediatric care. In Japan, similar to other countries, pediatricians often continue seeing patients with childhood-onset neurologic conditions without HCT. Some patients overlap with children with medical complexity and often see their health deteriorate due to the progression of underlying disease or complications when they become adults, hindering transfer to adult care.^19^ Clinic doctors were more likely to report fewer patients with childhood-onset neurologic conditions and experience more communication difficulties than those in hospitals. They also have fewer opportunities to treat adults with childhood-onset neurologic conditions.

Doctors with various specialty backgrounds cared for adults with childhood-onset neurologic conditions, which may relate to the fact that these people have multiple complications (Table 2, Table 3). However, it should be noted that the boundary between primary care physicians and specialists is unclear in Japan. Most doctors receive organ-specific training in their early career period. Then, once their training period is over, some doctors open their clinics and perform primary care within their specialty.^20^

### 4.1 The crucial role of HCPs in community- and hospital-based primary care for adults with childhood-onset neurologic conditions

In this study, HCPs agreed that for adults with childhood-onset neurologic conditions, having a primary care doctor is important. Simultaneously, HCPs have found it worthwhile to treat these patients, especially those with rare diseases and disabilities, and have valuable opportunities to enhance their medical experience and gain a broader perspective.

We observed that doctors and nurses played various roles in caring for adults with childhood-onset neurologic conditions, as previously reported.^10^ In addition to regular consultation, approximately half of the doctors and nurses provide patient education, which has been identified as an essential role of HCPs in primary care practice.^21^ All psychiatrists in this study were involved in patient education. Doctors with almost all specialties involving patient education might suggest the importance of self-management strategies such as preventable health conditions and the utilization of health and welfare services for the well-being of patients and families.

Primary care professionals’ involvement in HCT is desirable because HCT requires multifaceted support in terms of experience, health maintenance, and health and social service utilization.^22^ However, young patients and their families do not consider it necessary to see a primary care doctor because they are unaware of the importance of continuity of care or the role of the primary care doctor as a coordinator.^23^, ^24^ Therefore, HCPs who care for patients as specialists should educate patients and their families on the role of primary care doctors.

### 4.2 Challenges and barriers in caring for adults with childhood-onset neurologic conditions for HCPs

We listed examples of childhood-onset neurologic conditions such as cerebral palsy, epilepsy syndrome, Down syndrome and other chromosomal abnormalities, intellectual disability, and autism spectrum disorder in the questionnaire. Not all patients with such conditions have intellectual disabilities, but these conditions often overlap with intellectual disability.^8^ Many of the challenges HCPs experienced in treating adults with childhood-onset neurologic conditions in this study were similarly addressed as barriers to treating people with intellectual disabilities.^13^ To cope with “difficulty in communicating with patients,” HCPs can allow the patient to speak and learn “triadic communication,” similar to communication between a child, parent, and medical professional without ignoring the patient. In addition, HCPs can provide an independent representative for the patient and establish a multidisciplinary meeting involving healthcare professionals from various departments concerned with patient care to seek the best care plan for the patients.^25^, ^26^

The HCPs expressed concern about the “undecided emergency policy,” suggesting that decision-making for people with intellectual disabilities is challenging. However, primary care doctors can potentially play an important role in decision-making because they know the lives of their patients and their families well and utilize effective communication and advocacy skills to support decision-making for people with intellectual disabilities.^27^ Surveys on families of patients with intellectual disabilities indicate that families also recognize the importance and difficulty of preparing for the future.^28^ Patient education in conjunction with the daily medical care provided by HCPs enhances the decision-making capacity of patients and their families.

As indicated previously, the HCPs recognized the importance of communication and care for patients and their family members. They paid “special attention to the family’s feelings” because families and other caregivers of people with intellectual disabilities often experience intense stress.^29^ HCPs were often “overwhelmed with the demands of the family” when they required more resources and training for managing patient and family needs. Furthermore, emotional labor in dealing with various complex issues as front-line workers can influence the mental health of HCPs.^30^ Therefore, training targeting HCPs can include mental self-care for health professionals.^31^, ^32^

Our results indicated that HCPs exhibited apprehensions about managing complications outside their specialty and dealing with emergencies. Therefore, a system is required to facilitate primary care doctors’ referral of patients to other specialists. It is difficult to determine the extent to which specialists in the adult department and HCPs are involved in patient care,^33^ and there are differences in opinion on the appropriate level of involvement of primary care doctors and neurologists, even in common neurological diseases such as Parkinson’s disease or dementia, hindering the realization of coordinated care.^34^ Improved communication between primary care doctors and neurologists is desired for co-management.^35^ Further studies are needed to improve the availability of expert opinions and estimate their effectiveness.^36^, ^37^ Thus, as it is demanding to treat adults with childhood-onset neurological conditions, we need to investigate the impact of time and compensation on the care for these people to ensure the sustainability of the practice.

### 4.3 Desired system for pediatric-to-adult HCT

In line with recommendations and results from previous reports,^2^, ^38^ most HCPs supported “transition to adult care after a certain period of concurrent pediatric and adult care.” To achieve this, incentives for both the pediatric and adult departments are necessary. Furthermore, multidisciplinary conferences, which nurses highly desire, can be an excellent opportunity to learn how to manage childhood-onset conditions in other professions and establish face-to-face relationships. HCPs should also be financially guaranteed to provide proper care for the patient’s life and family.^39^ There is a high need for facilities for emergency hospitalization because any deterioration in their health condition can quickly lead to serious illness.

### 4.4 Limitations of this study

This study has some limitations. First, we asked health professionals currently caring for adults with childhood-onset neurological conditions to analyze HCP experiences. However, if we had included HCPs who are not presently involved in the care of this population, we might have collected a broader range of perspectives on the potential barriers and facilitators of HCT. Second, this study has a small sample size, particularly when considering the subsets of views discussed here (e.g., by profession or practice location), especially with limited presentation of nurses, therapists, care workers, and medical social workers who are involved in primary care for this population. Further research is needed to obtain insight from these HCPs directly, especially nurses. Third, this study mainly targeted HCPs working in three Tokyo cities; therefore, this study’s results cannot be generalized to other areas. Finally, we used self-reported questionnaires without additional validation testing besides content validity by a few physicians and with no initial pilot testing. Moreover, the results do not have any clear comparison or control group, and so it is difficult to say how the findings would compare to other patient populations. Additional research using validated questionnaires or concrete measurements such as electronic medical charts or insurance claims records may be required to clarify the detailed practices of HCPs.

## 5 Conclusion

We have described the roles and perspectives of HCPs involved in community- and hospital-based primary care for adults with childhood-onset neurological conditions. Multidisciplinary care of these adults, patient education to improve self-management and support decision-making, and care coordination of social resources are essential components of HCT. Although HCPs find it rewarding to care for these patients, it is critical to provide HCPs with more training opportunities specific to patients with childhood-onset neurologic conditions, including how to support people with disabilities and how to cope with patients’ multiple comorbidities and family needs. More efforts are needed to facilitate primary care in this population for better HCT.

## Funding statement

This work was partly supported by the Ministry of Health, Labour, and Wealth (Health and Labour Science Research Grants, Program Grant Number 21FC1015).

## Ethical statement

This study was approved by two Institutional Review Boards (M2020–168) and (2020–6). We obtained informed consent from all participants and anonymized their information for analysis.

## CRediT authorship contribution statement

**Mochizuki Yoko:** Conceptualization, Funding acquisition, Investigation, Methodology, Project administration, Resources, Supervision, Writing – review & editing. **Fujiwara Takeo:** Conceptualization, Formal analysis, Funding acquisition, Methodology, Project administration, Resources, Supervision, Writing – review & editing. **Osako Miho:** Conceptualization, Data curation, Formal analysis, Investigation, Methodology, Project administration, Resources, Software, Visualization, Writing – original draft, Writing – review & editing. **Yamaoka Yui:** Conceptualization, Formal analysis, Methodology, Software, Supervision, Validation, Writing – review & editing.

## Declaration of Generative AI and AI-assisted Technologies in the writing process

During the preparation of this work, the authors did not use AI or AI-assisted technologies.

## Declaration of Competing Interest

The authors declare that they have no known competing financial interests or personal relationships that could have appeared to influence the work reported in this paper.

## Data Availability

Data will be made available on request.
